# Advanced Heat Map and Clustering Analysis Using Heatmap3

**DOI:** 10.1155/2014/986048

**Published:** 2014-07-16

**Authors:** Shilin Zhao, Yan Guo, Quanhu Sheng, Yu Shyr

**Affiliations:** Center for Quantitative Sciences, Vanderbilt University, Nashville, TN 37232, USA

## Abstract

Heat maps and clustering are used frequently in expression analysis studies for data visualization and quality control. Simple clustering and heat maps can be produced from the “heatmap” function in R. However, the “heatmap” function lacks certain functionalities and customizability, preventing it from generating advanced heat maps and dendrograms. To tackle the limitations of the “heatmap” function, we have developed an R package “heatmap3” which significantly improves the original “heatmap” function by adding several more powerful and convenient features. The “heatmap3” package allows users to produce highly customizable state of the art heat maps and dendrograms. The “heatmap3” package is developed based on the “heatmap” function in R, and it is completely compatible with it. The new features of “heatmap3” include highly customizable legends and side annotation, a wider range of color selections, new labeling features which allow users to define multiple layers of phenotype variables, and automatically conducted association tests based on the phenotypes provided. Additional features such as different agglomeration methods for estimating distance between two samples are also added for clustering.

## 1. Introduction

Gene expression analysis is one of the most popular analyses in the field of biomedical research. In the age of high-throughput genomics, microarray technology dominated the market of high-throughput gene expression profiling for over a decade until the introduction of RNA-seq technology. Regardless of which high-throughput gene expression profiling assay used, the heat map is one of the most popular methods of presenting the gene expression data. A heat map is a graphical representation of data where the individual values contained in a matrix are represented as colors. There are many variations of heat map such as web heat map and tree map. Here, we focus on the biology heat map, which is typically used to represent the level of expression of genes across a number of comparable samples. A gene expression heat map's visualization features can help a user to immediately make sense of the data by assigning different colors to each gene. Clusters of genes with similar or vastly different expression values are easily visible. The popularity of the heat map is clearly evidenced by the huge number of publications that have utilized it.

Cluster analysis is another popular method frequently used with gene expression study [1]. In our context, clustering refers to the task of grouping together a set of samples based on the similarity of their gene expression patterns. There are two major applications of cluster analysis. First, it is often used as a quality control measurement for identifying outliers. Second, it can be used to classify sample subtypes. The majority of the time in gene expression studies, gene expression is quantified from samples originating from multiple biological conditions. For example, most gene expression studies will consist of disease and control groups. Samples are selected based on their phenotype. In the ideal scenario, after performing the cluster, samples with a specific phenotype are in one cluster and samples without this phenotype are in another cluster. However, in the real world, many factors can affect the cluster results. For example, biological contamination can cause a sample to fail to cluster within the group. Also, the phenotype used to select the sample might not be the driving force in this sample's gene expression pattern. There may be other phenotypes that cause the sample's gene expression pattern to behave differently from other samples within the same group. Thus, cluster analysis is an ideal tool to detect outlier samples in gene expression studies [2]. Also, cluster analysis can be used to identify novel subtypes [3]. For example, the breast cancer study from The Cancer Genome Atlas (TCGA) project [4] used clustering techniques to discover the subtype of samples based on their gene expression patterns. This is especially useful when subtypes of the samples are unknown. Also, the clustering technique can be applied to both sample and gene. When applied to both, the heat map can help visualizing potential novel pathways [5] and coexpression patterns [6].

The most popular tools to generate heat maps and clusters include the “heatmap” function in R and Cluster 3.0 [7]. However, these tools have some limitations. First, they can be slow and sometimes not able to finish for large expression matrices. Second, they are insufficient for producing advanced graphics. Third, they lack customizability. For example, in the breast cancer study from The Cancer Genome Atlas (TCGA) project [4] mentioned previously, the authors used heat map and cluster figures to present subtypes of the samples. The heat map used in that publication showed several additional bars to indicate phenotypes, and these phenotype bars are the result of meticulous work done by hand. A tool that can automatically display such phenotypes with the heat map is highly desirable. Driven by such motivation, we have produced “heatmap3,” an advanced heat map and cluster analysis tool in R. Our “heatmap3” package significantly improves the original “heatmap” function's functionality by adding more powerful and convenient features including highly customizable legends, multiphenotype display bars including continuous phenotypes such as age, a wider range of color selection, a wider range of distance and agglomeration method selection, and automatic association tests of phenotype and cluster groups. Our “heatmap3” package allows users to generate heat maps and clusters and to make annotations easily. Users with basic skill in R can operate “heatmap3” without trouble.

## 2. Implementation

The “heatmap3” package is developed based on the “heatmap” function in R, and it is also backward compatible with it (i.e., if a code were written for the “heatmap” function, it will also run with the “heatmap3” package without problem). All the commands and parameters for “heatmap” can also be used in “heatmap3.” We have implemented many new parameters in the “heatmap3” package in order to accommodate for the more powerful features. Detailed explanations and a manual of these parameters can be found at the hosting website of “heatmap3” (http://cran.r-project.org/web/packages/heatmap3/index.html).

### 2.1. Compute the Hierarchical Clustering between Rows and Columns

To assess the similarity of gene expression patterns between two samples, a distance or score needs to be computed. The original “heatmap” function used the Euclidean distance as the default distance method and complete linkage as the agglomeration method; it is not easy to change the default distance method within the original “heatmap” function. Our “heatmap3” package provides a wide selection of distance and agglomeration options, such as centered Pearson correlation, uncentered Pearson correlation, and average linkage. More importantly, “heatmap3” uses the clustering function in the “fastcluster” package when the expression matrix is large. This package efficiently implements the seven most widely used clustering schemes: single, complete, average, weighted, Ward, centroid, and median linkage. By using the “fastcluster” package, “heatmap3” is able to produce hierarchical clusters much faster and more efficiently than the original “heatmap” function.

### 2.2. Plot the Heat Map and Dendrogram

The “heatmap3” package sorts the rows and columns based on the hierarchical clustering result. The colors will then be assigned to the genes to represent the expression value. A balance option is provided here to ensure the median color will represent zero value. The heat map and dendrogram are plotted in the same fashion as the original “heatmap” function. However, more customization parameters are implemented. For example, the user now can choose to display or hide the dendrogram.

### 2.3. Plot the Color Bar, Annotation, and Legend

A color bar which represents the relationship between colors and values will be automatically generated at the top left side of the figure. The categorical phenotypes such as gender and race and the continuous phenotypes such as age and drug dose can be annotated in the column side of the heat map figure. This allows users to easily compare the annotation with the heat map results and make proper inference. Furthermore, “heatmap3” provides the function interfaces for generating the user's own annotations and legends. Users can use their own R functions to generate figures in the legend position and annotation position.

### 2.4. Cut and Statistically Test for Annotation in Different Groups

Our “heatmap3” package provides an automatic grouping method. A cutoff needs to be provided, and the dendrogram tree will be cut at the height of cutoff. The samples will be divided into several groups and labeled by different colors at the cutoff level. Then, statistical tests will be performed to see if the annotations are distributed equally in different groups. We used a chi-squared test for factor annotations and ANOVA for continuous annotations. These group results and *P* values will be returned to the user so that they can be used as criteria for selecting the genes that best separated the samples.

## 3. Results

To demonstrate the “heatmap3” package's efficiency and visualization power, we used RNA-seq gene expression results from the TCGA breast cancer (BRCA) dataset. The example dataset and its command can be downloaded from https://github.com/slzhao/heatmap3. The complete read count and clinical information can be seen in Tables S1 and S2 (see Supplementary Tables S1 and S2 available online at http://dx.doi.org/10.1155/2014/986048). To install the “heatmap3” package, type the following command in R:

 install.packages(“heatmap3”)

First, we performed differential analysis by the “edgeR” [8] package to compare the gene expression between triple negative samples versus nontriple negative samples. The *P* values and fold changes for genes were taken as annotation information (Table S3). We selected 500 genes with the largest standard deviations and randomly selected 30 samples to generate the heatmap. By selecting genes with large standard deviations, we effectively removed the nonexpressed genes across all samples, and the results still remained unbiased. We also included several important clinical variables for demonstration purposes. The selected phenotype variables were age, triple negative (TN) status, estrogen receptor (ER) status, progesterone receptor (PR) status, and human epidermal growth factor receptor 2 (HER2) status.

**Figure 1:**
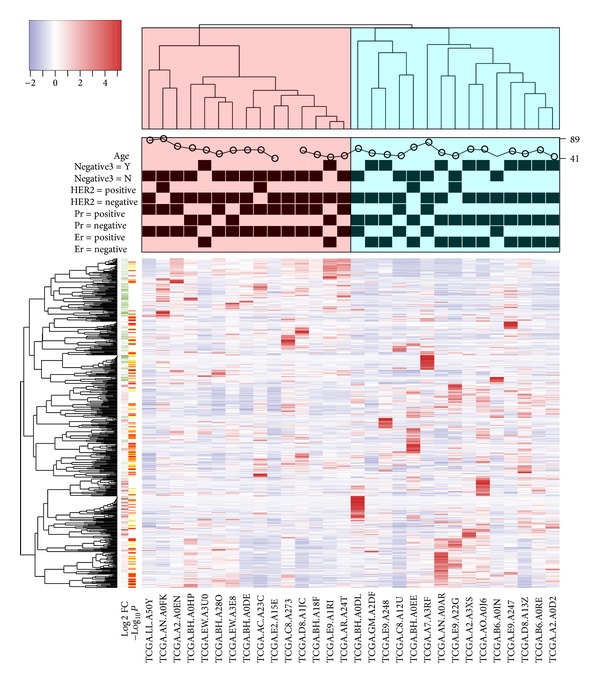
An example of “heatmap3” package. The heat map was generated based on 30 samples from TCGA BRCA dataset. The dendrogram of samples (top) was divided into two parts based on the correlation between samples' gene expression and then labeled, respectively. The categorical annotation bars (above heat map) demonstrate the annotation for age, TN, HER2, PR, and ER. The color bar on the left side demonstrates the log2 fold changes and negative log10 *P* values from comparison of triple negative patients versus nontriple negative patients.

Using these data, a heat map with legend color bar, column side annotations, and row side annotations was generated (Figure 1). The legend color bar indicates the relation between scaled expression values and colors, and the colors were balanced to ensure the white color represented zero value. We provided two annotation methods: color bar and categorical bar. Color bar is ideal to represent multiple phenotypes that are mutually exclusive. For example, for phenotypes of disease and normal, a sample can only be disease or normal but not both. Categorical bar is ideal to represent multiple phenotypes that are not mutually exclusive. For example, a sample can be TN and ER negative simultaneously.

The annotation on the *y*-axis side demonstrates how the customized function can be used for annotation. Here, we used the “showAnn” function within the package as an example. The categorical phenotype annotations (ER, PR, HER2 and TN) were separated into two columns, and the samples were labeled by black squares. The numeric annotation (age) was demonstrated by a scatter plot, and the values were labeled at the right axis. The annotation on the row side indicated an example of annotation by color bar. The green to red and orange to white colors here represent the log2 fold changes and the negative log10 *P* values, respectively. We can easily find that the genes increased in tripe negative samples (red color in log2 fold change annotation) were clustered in the bottom of heat map, while the genes decreased in tripe negative samples (green color in log2 fold change annotation) were clustered in the top of the heat map.

Using a height cutoff of 0.85 for the dendrogram tree on the column side, clearly, the samples were divided into two groups and labeled by different colors. As expected, the triple negative samples were enriched in the right group and nontriple negative samples were enriched in the left group. For the ER, PR, and HER2 levels, we can find that most of the samples were HER2 negative, and the ER and PR negative samples were enriched in the right group. Based on the results from the heat map, we might able to infer that ER and PR positive appear more in patients and they may have more important roles in defining triple negative samples. To generate Figure 1 using example data, enter the following command in R:

 # assume “counts” is the expression data, “colGene” contains the colors indicating fold changes and *P* value, and “clinic” contains the ER, PR, HER2, TN, and age information,
 temp**<-**apply(counts,1,sd),
 selectedGenes**<-**rev(order(temp))[1:500],
 heatmap3(counts[selectedGenes,],labRow=“”,margin =c(7,0),RowSideColors=colGene[selectedGenes,], ColSideCut=0.85,ColSideAnn=clinic,ColSideFun=** function**(x) showAnn(x),ColSideWidth=1.2,balance Color=T).

Association tests between phenotype and cluster groups were performed automatically by “heatmap3” (Tables 1 and 2). The number of categorical phenotypes and quantiles of continuous phenotype variables in each cluster group are summarized and reported. Chi-square test for categorical variables and ANOVA for continuous variables are performed by “heatmap3.” Based on the results, ER, PR, and HER2 were not equally distributed between the two clusters. On the other hand, age had no association with the two clusters (*P* = 0.429).

**Figure 2:**
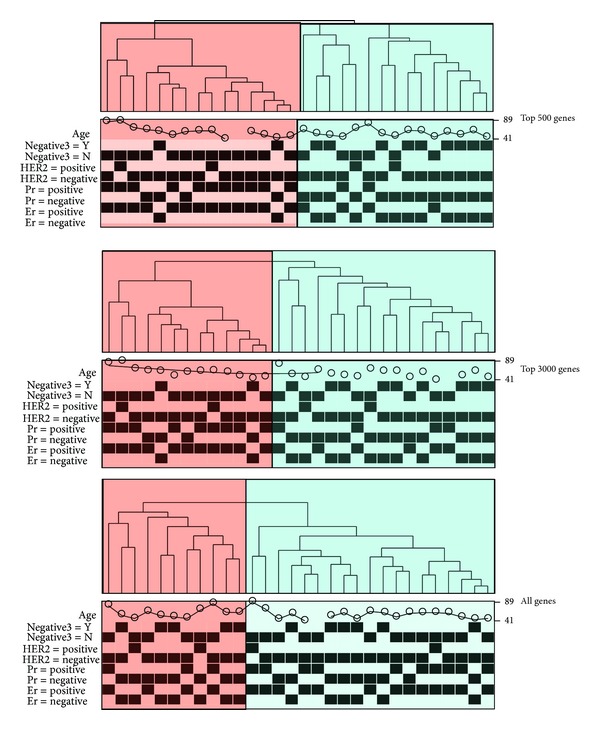
The dendrograms and clusters generated by top 500, top 3000, and all genes which were selected by standard deviation. The triple negative samples were more enriched in one group when genes with larger standard deviation were used. The results demonstrate that the “heatmap3” package can be helpful in selecting genes that best represent the phenotypes of samples.

The “heatmap3” package also provides an option which allows the generation of multiple heat maps and dendrograms based on the threshold criteria selected by the user. Using the same dataset, we performed heat map and cluster analysis using all genes, the top 3000 genes, and the top 500 genes selected by standard deviation.  Figure 2 shows the three dendrograms. All three dendrograms showed clearly two large clusters. Using TN status as the primary phenotype, each time a more stringent standard deviation cutoff was used, the clusters became clearer between TN and non-TN. This example illustrates the importance of selecting more statistically varied genes for subtyping purposes. We can conclude that the genes with the highest standard deviations can be used to separate the triple negative and nontriple negative samples. To generate Figure 2 using the example dataset, type the following commands in R:

 # assume “counts” is the expression data, “colGene” contains the colors indicating fold changes and *P* value, and “clinic” contains the ER, PR,HER2, TN, and age information,
 heatmap3(counts,topN=c(500,3000,nrow(counts)), labRow=“”,margin=c(7,0),RowSideColors=colGene, ColSideCut=0.85,ColSideAnn=clinic,ColSideFun=** function**(x) showAnn(x),ColSideWidth=1.2,balance Color=T).

## 4. Discussions

In this paper, we discussed the importance of heat map and clustering analysis as well as the limitations of existing heat map and clustering tools. To address these limitations, we implemented the “heatmap3” package in R and demonstrated its effectiveness using RNA-seq data from a breast cancer study in TCGA. The “heatmap3” package is designed with advanced options and is completely backward compatible with the original “heatmap” function in R. Users with limited R skill can generate sophisticated heat maps and dendrograms with ease. In summary, the “heatmap3” package fills the void of advanced graphical options in current heat map tools. It provides the much needed customizability for heat map and cluster analysis.

## Supplementary Material

Supplementary Material contains raw data to generate the figures in this paper. Table S1 contains the clinic information of the 30 TCGA BRCA samples, including the indicator of triple negative and age; Table S2 contains the read counts of genes in 30 TCGA BRCA samples; Table S3 contains the genes' fold changes and FDR, which were the result of comparison between triple negative and non triple negative samples.

## Figures and Tables

**Table 1:** The statistical test result for categorical annotation in different groups.

	Cluster1	Cluster2	*P* value by chi-square test
ER			
Negative	2	11	0.003
Positive	13	4	
Positive Percent	0.87	0.27	
PR			
Negative	4	13	0.003
Positive	11	2	
Positive Percent	0.73	0.13	
HER2			
Negative	13	13	0.023
Positive	2	2	
Positive Percent	0.13	0.13	

**Table 2:** The statistical test result for age in different groups, ANNOVA *P* value: 0.429.

Age	Cluster1	Cluster2
Min.	41.00	46.00
1st Qu.	51.00	49.00
Median	61.00	55.00
Mean	60.00	57.13
3rd Qu.	64.25	62.50
Max.	89.00	80.00

## References

[1] Guo Y, Zhao S, Ye F, Sheng Q, Shyr Y (2014). MultiRankSeq: multiperspective approach for RNAseq differential expression analysis and quality control. *BioMed Research International*.

[2] Yang S, Guo X, Yang Y (2006). Detecting outlier microarray arrays by correlation and percentage of outliers spots. *Cancer Informatics*.

[3] Sadanandam A, Lyssiotis CA, Homicsko K (2013). A colorectal cancer classification system that associates cellular phenotype and responses to therapy. *Nature Medicine*.

[4] Cancer Genome Atlas Network (2012). Comprehensive molecular portraits of human breast tumours. *Nature*.

[5] Lee JM, Sonnhammer ELL (2003). Genomic gene clustering analysis of pathways in eukaryotes. *Genome Research*.

[6] D'Haeseleer P, Liang S, Somogyi R (2000). Genetic network inference: from co-expression clustering to reverse engineering. *Bioinformatics*.

[7] Eisen MB, Spellman PT, Brown PO, Botstein D (1998). Cluster analysis and display of genome-wide expression patterns. *Proceedings of the National Academy of Sciences of the United States of America*.

[8] Robinson MD, McCarthy DJ, Smyth GK (2010). edgeR: a Bioconductor package for differential expression analysis of digital gene expression data.. *Bioinformatics*.

